# Culture, neuroscience, and law

**DOI:** 10.3389/fpsyg.2014.01196

**Published:** 2014-10-22

**Authors:** Gerardo Salvato, Roy Dings, Lucia Reuter

**Affiliations:** ^1^Department of Brain and Behavioral Sciences, University of PaviaPavia, Italy; ^2^Department of Psychiatry, Oxford Centre for Human Brain Activity, University of OxfordOxford, UK; ^3^Department of Philosophy, Radboud University NijmegenNijmegen, Netherlands; ^4^Department of Psychiatry, Charité University Hospital of BerlinBerlin, Germany

**Keywords:** cross-cultural differences, neuroscience, law and cognitive science, society, neuropsychological tests, neurolaw

## Society, neuroscience, and law

Nowadays, immigration flow to industrial cities has increased significantly. As a result, cultures are mixed and society has to deal with diverse languages, traditions and behaviors that appear to coexist in the same environment. This issue might influence the way justice is conducted. Legal trials increasingly involve citizens who have not necessarily grown up in the local culture or followed the same rules as native residents since their childhood. Interestingly enough, over the past few years, due to the growing complexity of legal trials, neuroscience and law started a promising partnership, which is now a recognized field of study (Goodenough and Tucker, 2010). Indeed, neuroscientific evidence in the courtroom offers reliable support in establishing the responsibility, free will and moral judgment of the defendant (Jones et al., 2013). When law calls for neuroscience in the courtroom, cross-cultural cases raise some ethical and practical concerns (Brickman et al., 2006). Mainly, these issues are related to the strong impact of culture on human behavior and to the absence of clear guidelines to follow when a neuroscientist is required to undertake a foreign defendant's profile assessment.

## The cultural shape of the human brain

The consistent modulatory and constitutional effects of culture on brain and behavior in humans have been largely demonstrated (for a review see Rule et al., 2013). For instance, differences in neural activity have been identified across cultures (Han and Northoff, 2008). It has been demonstrated that Americans show different brain activation patterns compared to Japanese people performing the same cognitive task on object processing during a functional Magnetic Resonance Imaging (fMRI) study (Gutchess et al., 2006). Behavioral studies exploring differences across populations have shown that culture has a considerable impact on basic cognitive functions, such as visual perception, memory and language. For instance, hunter-gatherers are less susceptible to the Müller-Lyer illusion (Segall et al., 1966). Moreover, evidence for differences in numerical cognition in several indigenous populations is present (Gordon, 2004; Pica et al., 2004; Frank et al., 2008). Culture could also influence spatial cognition (Majid et al., 2004). It has been shown that spatial cognitive strategies are modulated by the linguistic frame of references used in the native tongue. Indeed, language might influence the conceptual coding of space Pederson et al., 1998; Levinson et al., 2002; Haun et al., 2011. Cultural background also plays a pivotal role on more complex social mechanisms, such as emotion. In fact, the regulation of emotions is directly adjusted by culture, in which intrinsic norms guiding social interactions are present (De Leersnyder et al., 2013). For instance, Japanese people are more receptive than Dutch people in the vocal processing and perception of emotion (Tanaka et al., 2010).

## The transcultural cognitive assessment and its interpretation: some recommendations

As a result, anatomical and behavioral ethnic differences shaping social behavior might have some implications in the field of law. For example, previous studies have revealed that violent behaviors are more widespread in collectivistic compared to individualistic populations (Nesdale and Naito, 2005; Negy et al., 2013; Catalá-Miñana et al., 2014). More specifically, in the case of domestic violence, the severity of physical assaults was found to be higher in English than Spanish offenders (Catalá-Miñana et al., 2014). From this point of view, a comprehensive evaluation of the foreign defendant's profile could take into account these distinctive characteristics, which are relevant for both cognitive assessment and legal treatment. Remarkably, the neuroscientific assessment and legal report of foreign defendants raise a number of important questions. First of all, should tests that have specifically been developed for western subjects be used in the neuropsychological assessment of subjects of a different ethnicity? And if not, who is qualified to design and translate tests for ethnic minorities and non-English speakers? Who is qualified to administer and interpret the results? When a neuroscientist is required to undertake a legal report in the case of a foreign defendant's assessment, there are no clear guidelines to follow. In order to address these issues, we suggest an integrated approach accounting for cross-cultural differences that could be useful for a better understanding of the foreign defendants' profile in the courtroom. The assessment of cognitive functions through neuropsychological testing should include tests with adequate psychometric characteristics for culture or culture-free tests, in order to compare the subject's profile with a coherent group of healthy controls of the same age, education, gender and most importantly, the same cultural background. The importance of a qualitative as well as quantitative collection of data has been emphasized when neuropsychological tests are not available or scarce (e.g., few standardized neuropsychological measures for small-scale human communities) (Caetano, 2006). Indeed, a qualitative interpretation of results could shed light on the meaning of behaviors, choices and thoughts associated with a particular cultural background (Norenzayan, 2011). Additionally the involvement of a multidisciplinary team could be essential to more suitable assessments. Such a team could include experts belonging to both clinical and research practice. Hence, the “neuro-in-law” equip could involve at least a neuropsychologist, neuroscientist, neuroimaging expert, neurologist, psychiatrist, anthropologist as well as a translator to allow for a clear interpretation of the defendant's profile. Each of these experts could assess the defendant separately, with no prior knowledge of the diagnosis reached by other team members.

## Conclusions

**Figure 1:**
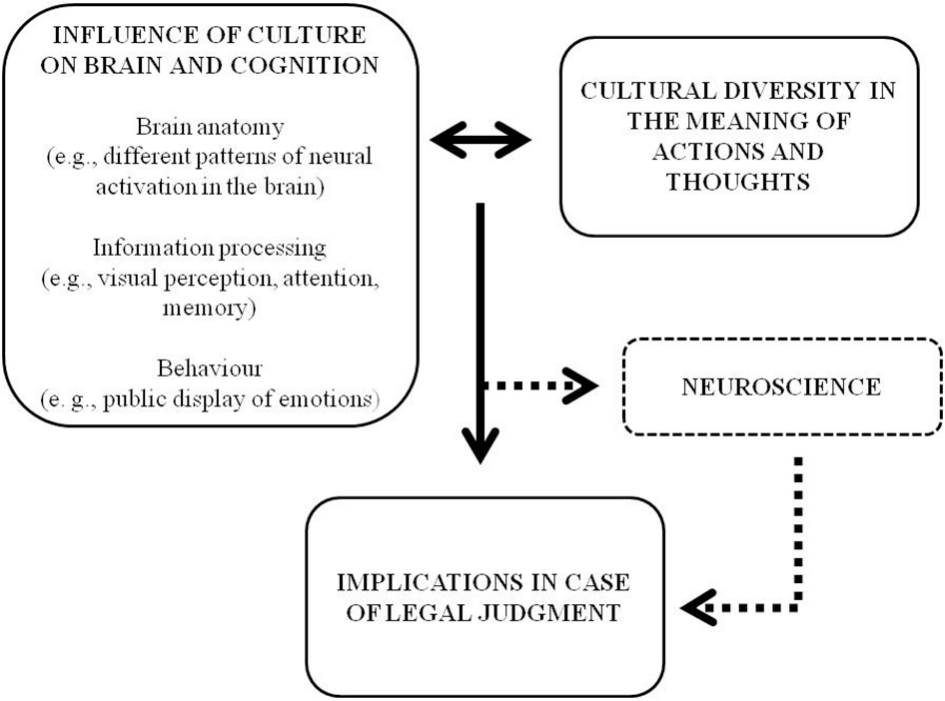
**Culture, neuroscience and law**. Culture influences human brain and cognition, hence the diversity we can observe in the meaning of actions and behaviors across different cultures. In the case of cross-cultural legal trials, this diversity could influence the legal outcomes. For example, the foreign defendants' or witnesses' testimonies might be influenced by the way they have perceived or remembered the situation, according to their cultural background. From this perspective, neuroscience could assist law for a clear and objective interpretation of the foreign defendant's behavior, taking into account the weight of culture on the brain.

Particular attention should be paid to the scientific methodology used for cognitive profiling when ethnic differences are present. Importantly, as the interaction between neuroscience and law is quite recent, is still necessary to establish suitable protocols for experts required to produce legal reports of foreign defendants. This issue is not marginal considering the influence of culture on behavior mentioned previously, as more and more legal trials also involve neuroscientific evidence. Additionally, it is worth noting that when cultural differences are taken into account, there is a risk that stereotypes leading to prejudice might be reinforced. For this reason, the cognitive diversity found across populations should be handled with some care as it might not be relevant to other fields of law (e.g., law enforcement official practice). Instead, when legal trials involve cultural diversity, a comprehensive neuroscientific procedure may contribute to more objective legal outcomes. In this case, neuroscience could assist law in decoding the significance of a range of culturally modulated social behaviors, which might have a strong impact on evidence examined in court (Figure 1). Advances in neuroscience are required to better fit the law's demands in the courtroom.

### Conflict of interest statement

The Associate Editor Asifa Majid declares that, despite being affiliated to the same institution as the author Roy Dings, the review process was handled objectively and no conflict of interest exists. The authors declare that the research was conducted in the absence of any commercial or financial relationships that could be construed as a potential conflict of interest.
